# Elucidating the role of TNF-like weak inducer of apoptosis in the pathogenesis of cutaneous lupus

**DOI:** 10.1186/ar4642

**Published:** 2014-09-18

**Authors:** Jessica Doerner, Jing Wen, Yumin Xia, Adam Friedman, Jennifer Michaelson, Linda Burkly, Lan Wu, Chaim Putterman

**Affiliations:** 1 Albert Einstein College of Medicine, Bronx, NY, USA; 2 Biogen Idec, Inc., Cambridge, MA, USA

## Background

Cutaneous manifestations are very common in systemic lupus erythematosus (SLE). TNF-like weak inducer of apoptosis (TWEAK) is a soluble cytokine member of the TNF superfamily that binds to a sole receptor, Fn14. TWEAK/Fn14 signaling is involved in cell survival, apoptosis, cytokine production, and angiogenesis, and as such has been found to be important in both tissue repair and inflammatory diseases. Whether TWEAK is involved in autoimmune skin disease is not known.

## Methods

To evaluate a possible role for TWEAK in the pathogenesis of cutaneous lupus, we generated a lupus-prone mouse strain, MRL-lpr/lpr (MRL/lpr), deficient in Fn14, and evaluated the development of skin disease in this strain as compared with age and gender-matched MRL/lpr mice. *In vitro *studies using a keratinocyte cell line, PAM212, were performed to evaluate the effects of TWEAK and ultraviolet B (UVB) irradiation on apoptosis and cytokine production.

## Results

We found that lupus-prone MRL/lpr mice deficient for Fn14 (Fn14KO) had markedly attenuated cutaneous disease as compared with Fn14WT mice. MRL/lpr Fn14KO mice had significantly less epidermal acanthosis and follicular plugging, and there was decreased infiltration of T cells and macrophages as compared with Fn14WT mice. *In vitro*, TWEAK treatment of murine keratinocytes stimulated the secretion of RANTES (via Fn14), and promoted apoptosis. Parthenolide decreased production of RANTES, indicating that this effect of TWEAK is mediated via NF-κB. Ultraviolet light, specifically UVB, is recognized as a potent trigger of cutaneous lupus. We found that TWEAK co-treatment exacerbates UVB light-induced keratinocyte apoptosis and increases the amount of RANTES secreted, when compared with cells treated with UVB alone. These synergistic effects of TWEAK+UVB on keratinocytes were probably due to upregulation of Fn14 expression by UVB.

## Conclusions

Our data strongly implicate TWEAK/Fn14 signaling in the pathogenesis of the cutaneous manifestations of lupus.

